# Different Predictors Shape the Diversity Patterns of Epiphytic and Non-epiphytic Liverworts in Montane Forests of Uganda

**DOI:** 10.3389/fpls.2020.00765

**Published:** 2020-06-24

**Authors:** Karola Maul, Yu-Mei Wei, Martin Nebel, Federico Luebert, Boon-Chuan Ho, Dietmar Quandt, Michael Kessler

**Affiliations:** ^1^Nees Institute for Biodiversity of Plants, University of Bonn, Bonn, Germany; ^2^Guangxi Key Laboratory of Plant Conservation and Restoration Ecology in Karst Terrain, Guangxi Institute of Botany, Guangxi Zhuang Autonomous Region and Chinese Academy of Sciences, Guilin, China; ^3^Departamento de Silvicultura y Conservatión de la Naturaleza, Universidad de Chile, Santiago, Chile; ^4^Singapore Botanic Gardens, National Parks Board, Singapore, Singapore; ^5^Institute of Systematic and Evolutionary Botany, Faculty of Mathematics and Natural Sciences, University of Zurich, Zurich, Switzerland

**Keywords:** epiphytic liverworts, non-epiphytic liverworts, species richness distribution, tropical montane forest, climatic predictors, Uganda

## Abstract

We studied the influence of regional and local variables on the liverwort diversity within natural forest vegetation of Uganda to contribute to our understanding of the mechanisms and processes determining species richness. To this end, we compared the species richness distribution patterns of epiphytic and non-epiphytic liverworts (Marchantiophytina) in 24 plots in the forests of four Ugandan national parks. We recorded a total of 119 species and subspecies from 18 families, including 16 new species records for the country. We used generalized linear models (GLMs) and the relative variable importance of regional and local climatic and environmental variables to assess their respective impact on the species diversity. We found that the richness patterns of total and epiphytic richness were largely driven by regional climatic factors related to temperature and water-availability. In contrast, species diversity of non-epiphytic and rare species was additionally strongly determined by local-scale microhabitat factors such as height of forest canopy and slope inclination, reflecting the availability of suitable microhabitats. We conclude that macroclimatic variables perform well in predicting epiphytic liverwort richness, whereas the adequate prediction of non-epiphytic richness requires site-specific variables. Also, we propose that richness of epiphytic liverworts will be impacted more directly by climate change than richness of non-epiphytic and rare species.

## Introduction

Biodiversity is unevenly distributed in space and time (Hawkins, 2001) and understanding the causes and drivers of its geographical distribution remains an essential goal in ecology and biogeography. Research has identified many different ecological and evolutionary drivers of biodiversity patterns, whose influence varies depending on the taxonomic group under consideration, the geographical region, and the spatial and temporal scales (e.g., Körner, 2000; McCain, 2005; McCain and Grytnes, 2010). Among these factors, climatic conditions have often been found to be closely correlated to contemporary species richness patterns (Araujo and Rahbek, 2006; Kessler et al., 2011). For instance, along elevational gradients, the balance between high temperatures and subsequent drought stress in the lowlands and low temperatures at high elevations may lead to optimal conditions and high diversity of many plant groups at mid-elevations, including ferns (Kluge and Kessler, 2011; Kluge et al., 2017) and bryophytes (Song et al., 2015). However, diversity can also be influenced by large-scale geographical factors such as land surface area (Karger et al., 2017) and geometric constraints in spatially restricted regions (Colwell et al., 2016). Besides these factors acting at broad spatial scales, diversity patterns are also influenced by localized factors, such as the presence of a specialized habitat required by a certain group of organisms. Although the importance of both regional- and local-scale factors is generally acknowledged (Crawley and Harral, 2001; Chalcraft, 2013), most studies identifying correlates of biodiversity patterns only focus on one of these scales, due to the availability of data at a given scale within a specific study. There is thus a need to combine factors affecting biodiversity at different spatial scales to better understand environmental drivers of biodiversity and to predict responses of biodiversity to changes in these drivers, e.g., as a result of climate change.

Bryophytes are an ecologically and evolutionarily distinct plant group including liverworts, mosses and hornworts, the almost globally distributed earliest extant land plant lineages. They are characterized by a poikilohydric lifestyle with a dominant haploid (gametophytic) and a short-lived diploid (sporophytic) generation. Because of their weak ability to actively regulate their water balance (Leon-Vargas et al., 2006; Proctor et al., 2007) bryophytes are poorly represented in arid regions, but in contrast are well adapted to low temperatures, so that they even occur in Antarctica and in high mountains above the treeline (Longton, 1982; Seppelt and Green, 1998; Bruun et al., 2006). It may thus not be so surprising that a global latitudinal gradient in species richness of mosses has not been supported (Shaw et al., 2005; Geffert et al., 2013). Liverworts in turn seem to be less frost tolerant than mosses (Glime, 2017), and a general latitudinal gradient in richness distribution has recently been demonstrated (Wang et al., 2017). Macroclimatic conditions including precipitation, temperature, and radiation have been found to be important drivers of these richness patterns (Sun et al., 2013; Zellweger et al., 2015; Spitale, 2016). Liverworts species richness, in particular, seems to be more closely correlated to macroclimate than moss richness (Aranda et al., 2014; Chen et al., 2015; Henriques et al., 2016).

On the other hand, bryophytes are small plants that are able to inhabit localized habitats like bare rocks and even leaves of vascular plants, when coupled with various strategies as well as physiological and structural adaptations (Kürschner, 2004; Kraichak, 2012). Hence, there are various studies showing a strong influence of small-scale biotic and abiotic habitat properties on different bryophyte species or growth forms, for instance relative air humidity and its daily fluctuations on the distribution of epiphyllous bryophytes in Costa Rica (Sonnleitner et al., 2009), vapor pressure deficit and soil moisture on growth of two moss species in Canada (Stewart and Mallik, 2006), and variations in canopy cover on species richness and species composition of terrestrial bryophytes in Ecuador (Mandl et al., 2009). Furthermore, it appears that the richness of epiphytic and non-epiphytic bryophytes may be influenced by a different set of factors, with the epiphytes being more closely linked to general climatic conditions (Gradstein, 2008; Zotz and Bader, 2009), and non-epiphytic species more closely to soil conditions or dead wood availability (Raabe et al., 2010). However, comparative studies researching the drivers of richness distribution patterns of epiphytic versus non-epiphytic liverworts in tropical forests are scarce. Several studies do not consider the ecological differences between mosses and liverworts (e.g., Sun et al., 2013), or do not distinguish between the sampled microhabitats (e.g., Grau et al., 2007; Tusiime et al., 2007). Other studies included only epiphytes (e.g., Wolf, 1993; Song et al., 2015) or terrestrials (e.g., Mandl et al., 2009), or focused on higher latitudes (e.g., Spitale, 2016).

In the present study, we compared the influence of regional-scale macroclimatic variables and local-scale factors such as relative air humidity, inclination, canopy closure, and canopy height on the species richness of epiphytic and non-epiphytic liverworts in Uganda. We addressed the following research questions:

(1) What is the relative importance of regional climatic factors and local site-specific factors for the prediction of species diversity patterns of Ugandan liverworts?
(2) Are there different factors determining the richness patterns of epiphytic and non-epiphytic liverworts?

## Materials and Methods

### Study Sites and Sampling Design

**FIGURE 1:**
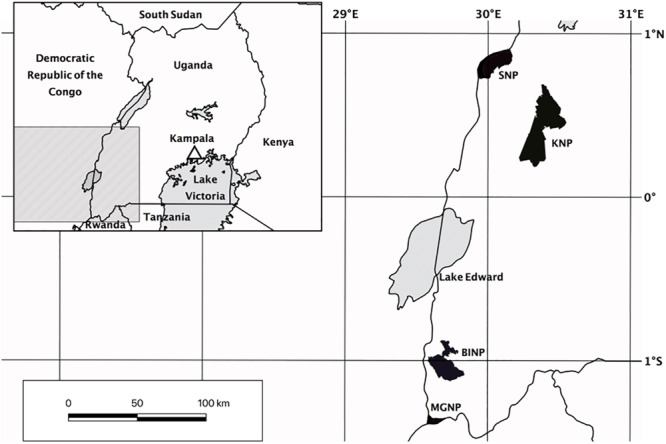
Map of study sites in Uganda. KNP, Kibale National Park; SNP, Semliki National Park; BINP, Bwindi Impenetrable National Park; MGNP, Mgahinga Gorilla National Park.

Because of the fragmented nature of forests in Uganda, we conducted our survey in four separate protected areas in western Uganda (Figure 1). Our first study site was the northern and middle parts of Kibale National Park (KNP), where we sampled five plots at 1270–1500 m. KNP is located on a plateau bordering the western Great Rift Valley. The low to high grade metamorphic bedrock belongs to the Paleoproterozoic Buganda-Toro System (Schlüter, 2006). The soil is either mostly low to moderately fertile consisting of red sandy loam, or fertile when overlying volcanic tuff (Howard, 1991). The vegetation in northern KNP consists of tropical moist evergreen forest up to 50 m tall with *Parinari excelsa* Sabine (Chrysobalanaceae) as one of the dominant tree species (Kasenene, 2001). *Olea welwitschii* Gilg & G.Schellenb. (Oleaceae), *Chrysophyllum* spp., *Aningeria altissima* (A.Chev.) Aubrév. & Pellegr. (Sapotaceae), *Strombosia scheffleri* Engl. (Olacaceae), *Newtonia buchananii* (Baker) G.C.C.Gilbert & Boutique (Fabaceae), *Celtis* spp. (Ulmaceae), *Diospyros abyssinica* (Hiern) F.White (Ebenaceae), and *Markhamia platycalyx* Sprague (Bignoniaceae) are characteristic for the mixed forest communities in the center of KNP.

We established three study plots at Semliki National Park (SNP), which is situated on the floor of the rift valley along Uganda’s border to the Democratic Republic of the Congo. The area is flat to hilly at 670–760 m, and largely covered with moist semi-deciduous forest (Howard, 1991). Although Uganda Ironwood (*Cynometra alexandri* C.H. Wright, Fabaceae) of up to 30 m tall is the dominating tree species, which partly forms almost pure stands (Howard, 1991), more than 300 tree species have been recorded within SNP in total (Howard et al., 2000). The larger part of the ground is poorly drained low fertility gray clay alluvials and rift sediments of recent to Neogene origin (Howard, 1991; Schlüter, 2006; Ring, 2008).

Our third study site was located in Bwindi Impenetrable National Park (BINP) in the Kigezi Highlands. This area has a rough terrain with steeply sloping mountains and narrow gorges that was shaped by the upwarping of the western rift valley. The groundrock is part of the Mesoproterozoic Karagwe-Ankolean System, which is low grade to unmetamorphosed (Schlüter, 2006). The soil is largely characterized by ferralitic humic loam, of low to high acidity and extremely poor in bases (Howard, 1991). BINP has an extremely rich flora and fauna, with many regionally endemic species (Butynski and Kalina, 1993). Large parts of BINP are occupied by mixed forest with *Prunus africana* (Hook.f.) Kalkman (Rosaceae), *Newtonia buchananii* (Baker) G.C.C.Gilbert & Boutique (Fabaceae), *Symphonia globulifera* L.f. (Clusiaceae), *Chrysophyllum* spp. (Sapotaceae), *Podocarpus* spp. (Podocarpaceae), and *Strombosia scheffleri* Engl. (Olacaceae) (Howard, 1991). We established 11 plots at 1515–2450 m in medium altitude moist evergreen forest and high elevation forest including two plots within the montane bamboo zone dominated by *Arundinaria alpina* K. Schum. (Poaceae) (Howard, 1991).

Finally, Mgahinga Gorilla National Park (MGNP) is the smallest part of the Virunga Conservation Area, which is situated on the border triangle between Uganda, Rwanda and the Democratic Republic of the Congo. MGNP covers the slopes of three volcanoes that are part of the Mesoproterozoic Karagwe-Ankolean System. The soils in the Virunga Massif are of volcanic origin and generally fertile (Hitimana et al., 2006; Akayezu et al., 2019). We established two plots at high altitude forest (2500–2800 m), including one within the broad zone of *Arundinaria* montane bamboo forest at the base of Mount Sabinyo (Langdale-Brown et al., 1964). Two plots were established in the following transition zone to the ericaceous forest at 2940 m and 3050 m, characterized by *Hypericum revolutum* Vahl (Hypericaceae) and *Hagenia abyssinica* J.F. Gmel. (Rosaceae). One plot was established in the ericaceous forest at 3200 m, which is characterized by *Philippia johnstonii* Schweinf. ex Engl. and *Erica arborea* L. (Ericaceae) (Owiunji et al., 2005).

In total, we established 24 plots of 20 × 20 m^2^ between 680 and 3200 m within mature natural forest, avoiding areas with vegetation disturbed by crossing trails and stream canyons. In each plot, we sampled eight subplots of 20 × 30 cm^2^ each, two for each of four microhabitats (soil, rotten logs, tree trunks above 1 m, and tree branches from 2 to 4 m). The subplots were located where bryophyte abundance was high. To obtain a plot inventory as complete as possible, we also sampled outside the subplots across the entire plot, including additional habitats (e.g., twigs, lianas, shrubs, small trees, tree bases etc.). We defined all specimens collected on tree trunks above 1 m, branches, twigs, lianas etc. as epiphytes. All specimens collected on the soil, and on rotten logs were assigned as non-epiphytes. Because tree trunk bases are a transition zone from non-epiphytic (soil, rotten logs, rocks) to epiphytic assemblages (Thomas et al., 2001; Holz et al., 2002), specimens occurring at the base of tree trunks (below 1 m), were treated as epiphytes when the majority of the other records of the species were epiphytic, or as non-epiphytic when the majority of other records was non-epiphytic. Species occurring in epiphytic and in non-epiphytic habitats were assigned to both habitats.

We used the checklist of Wigginton (2018) to assess the new records for Uganda, the literature cited therein was used as major reference to the species identification of the specimens (List S1). Classification and nomenclature followed Söderström et al. (2016). All vouchers are deposited in the herbarium of the Nees Institute for Biodiversity of Plants, University of Bonn (BONN), duplicates will be deposited in the herbarium of the Makerere University (MHU), Kampala, Uganda, after finishing the process of herbarium curation. Infraspecific taxa are treated as “species” for ease of data analyses and are simply regarded as such in the discussion.

### Data Analyses

**FIGURE 2:**
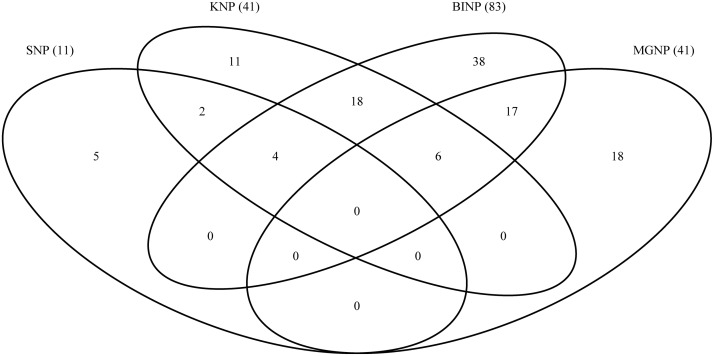
Venn diagram showing numbers of species shared and unique to the four study sites. SNP, Semliki National Park; KNP, Kibale National Park; BINP, Bwindi Impenetrable National Park; MGNP, Mgahinga Gorilla National Park. Numbers in brackets indicate the total species numbers recorded at each site.

The proportion of unique and shared species among the four national parks was assessed using a Venn diagram (Figure 2).

**FIGURE 3:**
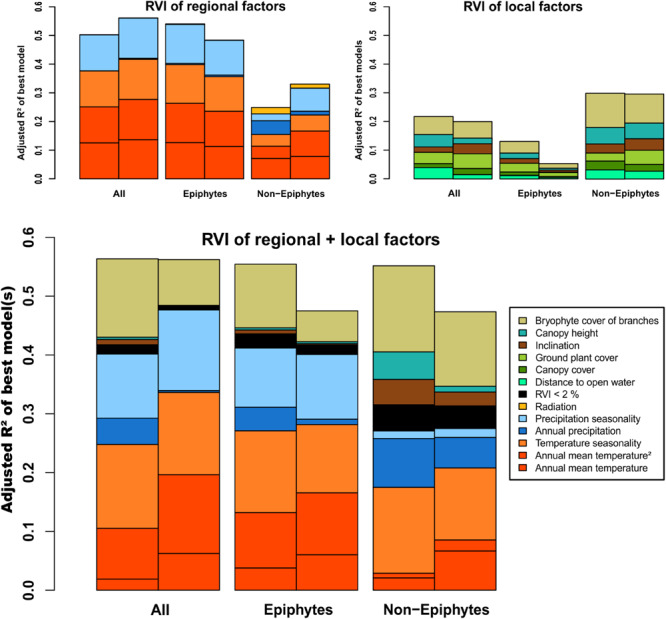
Barplots illustrating relative variable importance (RVI) of regional, local, and regional + local factors, relative to the according goodness of fit criterion (adjusted *R*^2^). Left bars show results from plot analyses, right bars from analyses restricted to subplot records.

We employed generalized linear models (GLMs) and two different parameter sets to assess the influence of climatic and environmental parameters on liverwort species richness. The regional dataset consisted of six climatic variables: annual mean temperature (Temp), temperature seasonality (TempS), annual precipitation (Prec), precipitation seasonality (PrecS), obtained from the CHELSA model (Karger et al., 2017), as well as solar radiation (Rad) obtained from Wilson and Gallant (2000) (Supplementary Table S1). Annual mean temperature was included as linear and quadratic term in the analyses (Temp^2^), since temperature might have a unimodal influence on richness. The local dataset included six variables recorded in each individual plot during fieldwork by visual estimation by always the same observer: inclination (Inc), percentage of ground covered by plants (GCov), canopy height (HC), canopy cover (CCov), percentage of canopy branch surface covered by bryophytes (BCov) as a proxy for air humidity (Karger et al., 2012), and distance to open water, which might also influence air humidity (DW; Supplementary Table S1). All 12 variables were associated among each other with pairwise Pearson’s correlation coefficients of ≤0.8 (Supplementary Table S2). We calculated GLMs with Poisson distribution and species richness as responding variables, and regressed them against all possible independent variable combinations from both parameter sets separately (regional: richness ∼ Temp + Temp^2^ + TempS + Prec + PrecS + Rad, local: richness ∼ Inc + GCov + HC + CCov + BCov + DW) and for the combined dataset (regional + local: richness ∼ Temp + Temp^2^ + TempS + Prec + PrecS + Rad + Inc + GCov + HC + CCov + BCov + DW). To avoid model overfitting, the possible combinations were restricted to a maximum of four variables per model in all the three approaches. The resulting models were ranked according to their respective second order Akaike information criterion (AICc) (Hurvich and Tsai, 1989). The goodness of fit of the best models (i.e., ΔAICc ≤ 2) was assessed using the Kullback–Leibler-divergence-based *R*^2^ (Cameron and Windmeijer, 1997). To check the significance of the improvement of the fitted best model compared to simpler models, we reduced the independent variables stepwise according to the lowest *z*-value and compared the change in deviance running chi-square tests (Table 1). The relative variable importance (RVI) of each single variable was calculated by summing up the AICc weights of all the models in which the respective variable was included (Figure 3 and Supplementary Table S3). To assess the effect of the additional non-standardized collections within the plots but outside subplots, we conducted the analyses including (a) all species records and (b) records from the subplots only (Figure 3 and Supplementary Tables S3, S4). Finally, we included the four to five (non-epiphytic richness) variables with highest RVI in univariate or polynomial (Temp^2^) GLMs to assess if the performance of the best models was a direct effect or part of a complex interplay between multiple variables (Supplementary Figure S1).

**TABLE 1:** Summary of the best models resulting from the regional (a), local (b), and combined regional + local dataset (c) within ΔAICc = 2.

**(a) Regional factors**	**(Intercept)**	**Temp**	**Temp^2^**	**Prec**	**PrecS**	**TempS**	**Rad**	**df**	**logLik**	**AICc**	**Delta**	**Weight**	**Adj. *R*^2^**	**Mean adj. *R*^2^**	**Pr(>Chi)**	
**All**	3.13***	−0.46***^4^	−0.42***^1^		−0.62***^2^	−0.64***^3^		5	−71.09	155.51	0	0.999	0.50		*/./***	
**Epiphytes**	2.87***	−0.36**^4^	−0.46***^1^		−0.59***^2^	−0.61***^3^		5	−62.99	139.31	0	0.915	0.54		./*/**	
**Non-epiphytes**	1.86***	−0.98***^1^		−0.76***^2^		−0.31*^3^		4	−68.22	146.54	0	0.168	0.25		***/*/–	

**(b) Local factors**	**(Intercept)**	**CCov**	**HC**	**DW**	**GCov**	**Inc**	**BCov**	**df**	**logLik**	**AICc**	**Delta**	**Weight**	**Adj. *R*^2^**		**Pr(>Chi)**	

**All**	2.75***		0.25**^2^	0.21**^3^	−0.12*^4^		0.33***^1^	5	−80.41	174.14	0	0.251	0.23		*/*/*	
	2.76***		0.29***^2^	0.19*^3^			0.30***^1^	4	−82.51	175.13	0.99	0.153	0.21	0.22	*/*/–	
**Epiphytes**	2.48***				−0.2**^1^	−0.14.^3^	0.29***^2^	4	−74.60	159.31	0	0.128	0.15		**/./–	
	2.48***				−0.19**^1^		0.2**^2^	3	−76.08	159.37	0.06	0.124	0.14		./–/–	
	2.47***		0.23*^3^	0.17.^4^	−0.15*^1^		0.26***^2^	5	−73.31	159.94	0.64	0.093	0.15		**/ /.	
	2.47***	−0.11^4^			−0.25***^1^	−0.16.^3^	0.37***^2^	5	−73.38	160.09	0.78	0.087	0.15		**/./	
	2.48***		0.10^3^		−0.14.^1^		0.21**^2^	4	−75.1	160.31	1.0	0.078	0.13		**/ /–	
	2.48***	−0.1^3^			−0.22**^1^		0.25**^2^	4	−75.17	160.44	1.13	0.073	0.13		**/ /–	
	2.47***		0.10^4^		−0.15*^1^	−0.14.^3^	0.31***^2^	5	−73.68	160.68	1.34	0.064	0.14		**/./	
	2.49***		0.17**^2^				0.19**^1^	3	−76.97	161.15	1.84	0.051	0.10		**/–/–	
	2.48***		0.28**^2^	0.15.^3^			0.23**^1^	4	−75.59	161.28	1.97	0.048	0.11	0.13	**/./–	
**Non-epiphytes**	1.85***		0.13.^2^				0.54***^1^	3	−67.19	141.58	0	0.15	0.31		./–/–	
	1.85***						0.49***^1^	2	−68.63	141.83	0.26	0.13	0.31		–/–/–	
	1.84***		0.27*^2^	0.18^3^			0.6***^1^	4	−66.18	142.46	0.88	0.1	0.31		./ /–	
	1.85***		−0.08^2^				0.53***^1^	3	−68.08	143.36	1.78	0.06	0.29		/–/–	
	1.85***					−0.1^2^	0.57***^1^	3	−68.11	143.42	1.84	0.06	0.29	0.3	/–/–	

**(c) Regional** + **local factors**	**(Intercept)**	**Temp**	**Temp^2^**	**Prec**	**PrecS**	**TempS**	**Inc**	**BCov**	**df**	**logLik**	**AICc**	**Delta**	**Weight**	**Adj. *R*^2^**	**Mean adj. *R*^2^**	**Pr(>Chi)**

**All**	2.94***		−0.23***^4^		−0.43***^3^	−0.75***^2^		0.61***^1^	5	−69.01	151.36	0	0.53	0.56		*/***/***
**Epiphytes**	2.72***		−0.3***^1^		−0.44***^2^	−0.7***^3^		0.46***^4^	5	−62.25	137.83	0	0.41	0.57		. /*/***
	2.87***	−0.35**^4^	−0.45***^1^		−0.59***^2^	−0.64***^3^			5	−62.99	139.31	1.48	0.19	0.54	0.55	. /*/**
**Non-epiphytes**	1.71***			−0.43***^3^		−1.09***^2^	−0.19*^4^	1.82***^1^	5	−56.13	125.59	0	0.19	0.57		*/***/*
	1.74***			−0.41***^3^		−0.98***^2^		1.53***^1^	4	−58.11	126.33	0.74	0.13	0.53	0.55	*/***/–

Temp, annual mean temperature; Temp^2^, (annual mean temperature)^2^; Prec, annual precipitation; PrecS, precipitation seasonality; TempS, temperature seasonality; Rad, solar radiation; CCov, canopy cover; HC, height of canopy; DW, distance to open water; GCov, ground plant cover; Inc, inclination; BCov, bryophyte cover of branches; significance of *Z*-value of the respective variable is given after each coefficient estimate. df, degrees of freedom; delta, difference AICc to the best model; adj. *R*^2^, adjusted *R*^2^; mean adj. *R*^2^, arithmetic mean of *R*^2^ of the best models within delta AICc = 2, asterisks after coefficient estimates indicate significance level of variable in the respective model (Pr(>| *z*|). Superscripts depict the order of *z*-value magnitude, with ^*1*^ = highest absolute z-value. Pr(>Chi) = significance of improvement of best models compared to simpler models (obtained by successive removal of least significant variables based on z-values). (–) = indicate best model with less than four variables. Significance codes: ***0 < *p* ≤ 0.001; **0.001 < *p* ≤ 0.01; *0.01 < *p* ≤ 0.05; (.) 0.05 < *p* ≤ 0.1.

All computational analyses were conducted in R 3.6.0 (R Core Team, 2017) using the packages VennDiagram 1.6.20 (Chen, 2018), MuMIn 1.43.6 (Barton, 2019), and rsq 1.1 (Zhang, 2018).

## Results

We obtained a total of 940 species-plot records of which 653 (69.5%) were collected within the subplots. Our collection included a total of 119 species, subspecies and varieties from 19 families. No fewer than 16 species were new records for Uganda (Table 2). The most species rich family was Lejeuneaceae (51 species) and the most speciose genera were *Lejeunea* (22) and *Plagiochila* (18). Out of all collected species *Plagiochila kiaeri* Gottsche and *Metzgeria furcata* (L.) Corda were recorded in the majority of plots (16 and 14 plots, respectively, Table 2). The species occupying the widest elevational range was *Lejeunea conformis* Nees et Mont. (9 plots, 1275–3200 m). On average, we recorded 16.4 species per plot (range 3–28). A total of 48 species occurred exclusively in epiphytic and 30 species in non-epiphytic habitats; 41 species were recorded from both habitat categories. Ninety seven species and subspecies (81%) were collected from within the subplots only.

**TABLE 2:** Liverwort inventory with collection sites and habitats.

**Plot**		**1**	**2**	**3**	**4**	**5**	**6**	**7**	**8**	**9**	**10**	**11**	**12**	**13**	**14**	**15**	**16**	**17**	**18**	**19**	**20**	**21**	**22**	**23**	**24**
**GPS-coordinates:**	**Latitude**	0.564722222	0.566083333	0.451680556	0.459275	0.642444444	0.829669444	0.833180556	0.837138889	−0.99268333	−0.99443611	−0.98994444	−0.98823333	−1.01033611	−1.00949722	−1.09643333	−1.08771389	−1.05808889	−1.07576667	−1.07199167	−1.38119722	−1.38446944	−1.38297222	−1.37603056	−1.37719444
	**Longitude**	30.3583278	30.3561389	30.3849361	30.3777111	30.3946667	30.0893778	30.0896806	30.0886083	29.614725	29.6144389	29.60675	29.6245861	29.7379222	29.7378611	29.8033	29.8080361	29.7954611	29.7641139	29.7677833	29.6026	29.5990806	29.6011222	29.6044972	29.6040833
**Site**		KNP	KNP	KNP	KNP	KNP	SNP	SNP	SNP	BINP	BINP	BINP	BINP	BINP	BINP	BINP	BINP	BINP	BINP	BINP	MGNP	MGNP	MGNP	MGNP	MGNP
**Date of collection**		15.Feb.2014	15.Feb.2014	16.Feb.2014	16.Feb.2014	17.Feb.2014	18.Feb.2014	18.Feb.2014	18.Feb.2014	23.Feb.2014	23.Feb.2014	23.Feb.2014	24.Feb.2014	25.Feb.2014	25.Feb.2014	26.Feb.2014	26.Feb.2014	27.Feb.2014	28.Feb.2014	28.Feb.2014	02.Mar.2014	02.Mar.2014	02.Mar.2014	03.Mar.2014	03.Mar.2014
**Exposition**		S	-	-	SW	E	-	-	-	E	E	W	NW	N	S	W	S	E	N	E	NE	N	N	N	E
**Inclination [°]**		5	0	0	5	8	0	0	0	5	10	15	25	45	40	15	25	20	20	15	15	45	35	5	20
**Altitude [m.a.s.l.]**		1500	1530	1270	1275	1450	700	680	690	1550	1514	1570	1700	1950	1980	2300	2450	2350	2100	2200	2940	3200	3050	2700	2750
**Species**	**Family**																								
*Pseudomarsupidium decipiens* (Hook.) Grolle	Adelanthaceae																					e, rl			
*Plicanthus hirtellus* (F.Weber) R.M.Schust.	Anastrophyllaceae													t											
*Aneura pinguis* (L.) Dumort.	Aneuraceae	rl									rl														
*Riccardia* sp. 1	Aneuraceae			rl*																					
*Riccardia* sp. 2	Aneuraceae									rl	t									t*					
*Riccardia* sp. 3	Aneuraceae							rl																	
*Riccardia* sp. 4	Aneuraceae									e*	rl*											rl*			rl*
*Calypogeia fissa* (L.) Raddi	Calypogeiaceae																								t
*Cylindrocolea abyssinica* (Gola) Váòa	Cephaloziellaceae																			rl					
*Dumortiera hirsuta* (Sw.) Nees	Dumortieraceae	t*				t*					t*														
*Frullania caffraria* Steph.	Frullaniaceae																		e*					e	
*Frullania ericoides* (Nees) Mont.	Frullaniaceae					e																			
																									
*Frullania obscura* (Sw.) Mont.	Frullaniaceae														e			e			e			e	
*Frullania obscurifolia* Mitt.	Frullaniaceae										e													e	
*Frullania trinervis* (Lehm.) Drège	Frullaniaceae	e	e	e	e	e	e	e		e			e												
*Haplomitrium* sp.	Haplomitriaceae																					t*			
*Herbertus dicranus* (Gottsche. Lindenb. et Nees) Trevis	Herbertaceae																					e, rl, t	e		
*Acanthocoleus chrysophyllus* (Lehm.) Kruijt	Lejeuneaceae	e	e																						
*Caudalejeunea lehmanniana* (Gottsche) A.Evans	Lejeuneaceae						e																		
*Caudalejeunea lewallei* Vanden Berghen	Lejeuneaceae									e	e	e	e		e										
*Ceratolejeunea cornuta* (Lindenb.) Steph.	Lejeuneaceae									e*		e													
*Cheilolejeunea krakakammae* (Lindenb.) R.M.Schust.	Lejeuneaceae													e*					e*						
*Cheilolejeunea montagnei* (Gottsche ex Mont.) R.M.Schust.	Lejeuneaceae													rl											
*Cheilolejeunea roccatii* (Gola) W.Ye. R.L.Zhu et Gradst.	Lejeuneaceae																					e, rl	e, rl		
*Cheilolejeunea surrepens* (Mitt.) E.W.Jones	Lejeuneaceae								e																
*Cheilolejeunea xanthocarpa* (Lehm. et Lindenb.) Malombe	Lejeuneaceae																			e					
*Cololejeunea elegans* Steph.	Lejeuneaceae										e*				e										
*Cololejeunea harrisii* Pócs	Lejeuneaceae										e*														
*Cololejeunea lemuriana* Tixier	Lejeuneaceae											e													
*Diplasiolejeunea runssorensis* Steph.	Lejeuneaceae														e, rl							e	e		
*Drepanolejeunea physifolia* (Gottsche) Pearson	Lejeuneaceae											e		e	e						e	e	e		
*Lejeunea abyssinica* (Gola) Cufod	Lejeuneaceae														e	e, rl									
*Lejeunea alata* Gottsche	Lejeuneaceae																							e	e*
																									
**Plot**		**1**	**2**	**3**	**4**	**5**	**6**	**7**	**8**	**9**	**10**	**11**	**12**	**13**	**14**	**15**	**16**	**17**	**18**	**19**	**20**	**21**	**22**	**23**	**24**
*Lejeunea anisophylla* Mont.	Lejeuneaceae	rl				e, rl	rl	e	e																
*Lejeunea aphanes* Spruce	Lejeuneaceae					e*				e, rl	e	e	e					e*							
*Lejeunea brenanii* E.W.Jones	Lejeuneaceae													e, b*											
*Lejeunea cantabrigiensis* E.W.Jones	Lejeuneaceae																		rl*						
*Lejeunea capensis* Gottsche	Lejeuneaceae																							e	
*Lejeunea conformis* Nees et Mont.	Lejeuneaceae	rl, t			e*					e*, rl, b*			e			rl			e, rl*, t*			rl*		e*	rl
*Lejeunea eckloniana* Lindenb.	Lejeuneaceae	e, rl		e		e		e				e													
*Lejeunea flava* (Sw.) Nees	Lejeuneaceae													e	e	e*		e, rl, b*		e, rl*				e	
*Lejeunea flava* ssp. *tabularis* (Spreng.) S.W.Arnell	Lejeuneaceae		e*		e					e*	e	e			e*										
*Lejeunea flavovirens* Ångstr.	Lejeuneaceae																e, rl*			e	e	e	e	e	
*Lejeunea ibadana* A.J.Harr. et E.W.Jones	Lejeuneaceae																								e
*Lejeunea isophylla* E.W.Jones	Lejeuneaceae																	e		rl	e, rl			e	e
*Lejeunea lomana* E.W.Jones	Lejeuneaceae		e							e						e	e, rl*	e, rl	e, rl		rl*			e	rl
*Lejeunea papilionacea* Prantl	Lejeuneaceae	e											e		e	e									
*Lejeunea phyllobola Nees & Mont.*	Lejeuneaceae	e	e																						
*Lejeunea ramosissima* Steph.	Lejeuneaceae									e*, rl*	e, rl	t		e, b*	e, rl										
*Lejeunea setacea* (Steph.) Steph.	Lejeuneaceae							e																	
*Lejeunea* sp. 1	Lejeuneaceae																			rl					
*Lejeunea tuberculosa* Steph.	Lejeuneaceae	e	e			e				e		e	e*		e	e			e						
*Lejeunea villaumei* (Steph.) Grolle	Lejeuneaceae												e*												
*Lopholejeunea revoluta* E.W.Jones	Lejeuneaceae	e*																							
*Marchesinia excavata* (Mitt.) Schiffn.	Lejeuneaceae									rl*															
*Marchesinia nobilis* (Gottsche) X.Q.Shi. R.L.Zhu et Gradst.	Lejeuneaceae									t															
*Mastigolejeunea* sp.	Lejeuneaceae		e																						
*Microlejeunea africana* Steph.	Lejeuneaceae		e									e		e				e						e	
																									
**Plot**		**1**	**2**	**3**	**4**	**5**	**6**	**7**	**8**	**9**	**10**	**11**	**12**	**13**	**14**	**15**	**16**	**17**	**18**	**19**	**20**	**21**	**22**	**23**	**24**
*Microlejeunea* sp. 1	Lejeuneaceae																			e					
*Microlejeunea* sp. 2	Lejeuneaceae										e														
*Microlejeunea ulicina* (Taylor) A.Evans	Lejeuneaceae																		e						
*Prionolejeunea grata (Gottsche) Schiffn.*	Lejeuneaceae										e	e													
*Ptychanthus africanus* Steph.	Lejeuneaceae	e*	e	e*	e	e, rl*				e, rl		e	e												
*Schiffneriolejeunea altimontana* Vanden Berghen	Lejeuneaceae													e	e					e, rl					
*Schiffneriolejeunea pappeana* (Nees) Gradst. var. *pappeana*	Lejeuneaceae		e							e	e*														
*Schiffneriolejeunea polycarpa* (Nees) Gradst.	Lejeuneaceae		e*							e*															
*Spruceanthus abbreviatus* (Mitt.) X.Q.Shi. R.L.Zhu et Gradst.	Lejeuneaceae			e	e		e	e	e*																
*Thysananthus auriculatus* (Wilson) Sukkharak et Gradst. var. *auriculatus*	Lejeuneaceae						e																		
*Bazzania decrescens* (Lehm. et Lindenb.) Trevis.	Lepidoziaceae													t*											
*Bazzania nitida* (F.Weber) Grolle	Lepidoziaceae											t		e, rl, t											
*Lepidozia cupressina* (Sw.) Lindenb. ssp. *cupressina* Pócs	Lepidoziaceae													rl											
*Lepidozia stuhlmannii* Steph.	Lepidoziaceae																				b*	rl			
*Lepidozia succida* Mitt.	Lepidoziaceae										t*														
*Telaranea coactilis* (Spruce) J.J.Engel et G.L.Merr.	Lepidoziaceae										rl*														
*Telaranea nematodes* (Gottsche ex Austin) M.Howe	Lepidoziaceae										t	rl	rl	t	t			t		rl*	t				
*Telaranea diacantha* (Mont.) J.J.Engel et G.L.Merr.	Lepidoziaceae										rl, t	rl, t													
*Cryptolophocolea martiana* ssp. *martiana* (Nees) L.Söderstr. Crand.-Stotl. et Stotler	Lophocoleaceae	rl	rl							rl, t	rl, t, b*	rl, t	rl	rl	rl					rl, t					
*Leptoscyphus infuscatus* (Mitt.) E.W.Jones ex Grolle	Lophocoleaceae																					e, rl		rl*	t
																									
**Plot**		**1**	**2**	**3**	**4**	**5**	**6**	**7**	**8**	**9**	**10**	**11**	**12**	**13**	**14**	**15**	**16**	**17**	**18**	**19**	**20**	**21**	**22**	**23**	**24**
*Lophocolea bidentata* (L.) Dumort.	Lophocoleaceae															rl, t		e, rl*	rl	rl	rl*	rl*	rl*	e, rl	rl, t*
*Lophocolea concreta* Mont.	Lophocoleaceae		rl															rl		rl					
*Lophocolea difformis* Nees	Lophocoleaceae					rl																			
*Lophocolea muricata* (Lehm.) Nees	Lophocoleaceae										e	rl		t	rl	t		t	rl	rl				rl*	t*
*Andrewsianthus bilobus* (Mitt.) Grolle	Lophoziaceae																					rl			
*Metzgeria consanguinea* Schiffn.	Metzgeriaceae					e													e, t*	e*	rl*	rl*		e	rl
*Metzgeria crassipilis* (Lindb.) A.Evans	Metzgeriaceae																	e						e*	
*Metzgeria furcata* (L.) Corda	Metzgeriaceae	e	e	e	e	e				e		e		e	e	t			e, rl	e				e	e
*Metzgeria madagassa* Steph.	Metzgeriaceae	e								e, rl			e*	e*	e, rl	e*		rl		e					
*Metzgeria quadrifaria* Steph.	Metzgeriaceae									e		e				e				e		e*			
*Pallavicinia lyellii* (Hook.) Gray	Pallaviciniaceae											t, b*													
*Plagiochila colorans* Steph.	Plagiochilaceae																					e, rl*			
*Plagiochila ericicola* Steph.	Plagiochilaceae																					rl*			
*Plagiochila fusifera* Taylor	Plagiochilaceae											e		e, t	e, rl										
*Plagiochila heterostipa* Steph.	Plagiochilaceae																				e*				
*Plagiochila integerrima* Steph.	Plagiochilaceae	rl, t		t*		rl*				rl	rl	e, rl*	e, rl*												
*Plagiochila kiaeri* Gottsche	Plagiochilaceae	e, t	e	e	e	e, rl				e, rl, t	e, rl, t		e		e, rl	e, rl, t	e	e*, rl	e	e, rl, b*				e, b*	e, t*
*Plagiochila kiaeri* var. *myriocarpa* (Pearson) Pócs	Plagiochilaceae		e									e	e*	e*	e										
*Plagiochila lastii* Mitt.	Plagiochilaceae											e, rl		e*	e				t*, b*	e, rl					
*Plagiochila pectinata* Lindenb.	Plagiochilaceae																		e						
*Plagiochila pinniflora* Steph.	Plagiochilaceae		e			e*, rl*																			
*Plagiochila* sp. 1	Plagiochilaceae			rl*	e*		rl*																		
*Plagiochila* sp. 3	Plagiochilaceae																						e *		
*Plagiochila* sp. 4	Plagiochilaceae																							rl *	
*Plagiochila squamulosa* Mitt.	Plagiochilaceae																				e*			e, rl	e*, rl
*Plagiochila squamulosa* var. *crispulo-caudata* (Gottsche) Vanden Berghen	Plagiochilaceae										t														
*Plagiochila squamulosa* var. *sinuosa* (Mitt.) Vanden Berghen	Plagiochilaceae															rl		e, rl, b*		e, rl	e, b*			e, rl	e, rl
																									
*Plagiochila strictifolia* Steph.	Plagiochilaceae	e, rl		e	rl	e, rl*	e	e	e	e, rl*, t			e												
*Plagiochila terebrans* Nees et Mont.	Plagiochilaceae		e			e				e	e, rl, t	e	e*	e, t	e				e, b*						
*Porella abyssinica* var. *hoehnelii* (Steph.) Pócs	Porellaceae	rl				e																			
*Porella capensis* (Gottsche) Mitt.	Porellaceae										e														
*Porella subdentata* (Mitt.) Steph. var. *subdentata*	Porellaceae	e	e, t	e, rl	e, rl	e, rl*					e														
*Porella subdentata* var.*camerunensis* E.W.Jones	Porellaceae									e*															
*Radula ankefinensis* Gottsche	Radulaceae					e						e, rl			e					rl*					
*Radula boryana* (F.Weber) Nees ex Mont.	Radulaceae									e	e, rl	e, b*		e*											
*Radula flaccida* Lindenb. et Gottsche	Radulaceae	e				e	e	e*	e			e													
*Radula fulvifolia* (Hook.f. et Taylor) Gottsche. Lindenb. et Nees	Radulaceae	e		e						e, rl, b*	e, t			e											
*Radula quadrata* Gottsche	Radulaceae													e*							e				
*Radula stenocalyx* Mont.	Radulaceae					e*																			
*Radula voluta* Taylor	Radulaceae													e					e*, t*					e	
*Solenostoma borgenii* (Gottsche) Steph.	Solenostomataceae																					t*			
																									

*Sites: KNP, Kibale National Park; SNP, Semliki National Park; BINP, Bwindi Impenetrable National Park; MGNP, Mgahinga Gorilla National Park. Underlined species names indicate the first record of the taxon for Uganda. Habitats: t, terrestrial; e, epiphytic; rl, rotten log; b, tree base (below 1 m), asterisks mark records from outside subplots.*

60.5% of all species recorded were found in only one national park, and no species was found in all four national parks (Figure 2). 8.4% of the species were shared between three national parks, and 31.1% were found in two national parks. KNP shared 28 (68%) of its recorded species with BINP, and BINP and MGNP shared 23 species, whereas SNP and MGNP did not share any species.

The best models for the regional variables explained 50% of the variance in total species richness (Figure 3 and Table 1). Roughly half of the explained variance (25.1%), was accounted for by annual mean temperature (Temp + Temp^2^) followed by temperature seasonality (TempS; 12.5%) and precipitation seasonality (PrecS; 12.5%). Epiphytic species richness showed a very similar pattern, with 54% of the variance explained by the same factors in roughly similar proportions. Non-epiphytic species richness showed a different pattern, with 25% of variance explained, half of which was accounted for by annual mean temperature (Temp + Temp^2^; 11.4%), followed by annual precipitation (Prec; 4.8%), and temperature seasonality (TempS; 4%) (Figure 3).

The models considering local environmental variables alone had lower explanatory power than the regional variables for total and epiphytic richness, whereas the reverse was true for non-epiphytic richness (Figure 3 and Table 1). For total richness, the models explained 21.8% of the variance, with bryophyte cover of branches (BCov) as the most important contributor (6.3%), followed by canopy height (HC; 4.3%) and distance to open water (DW; ∼ 4%) as well as plant cover of ground (GCov; ∼ 4%). For epiphytic richness, variance explained was only 13.1%, with bryophyte cover of branches (BCov) and plant cover of ground (GCov) as main factors (4% and 3%, respectively). For non-epiphytic species richness, the models explained 29.9% of the variance, largely accounted for by bryophyte cover of branches (BCov; 11.9%) followed by canopy height (HC; 5.8%) (Figure 3).

Combining regional and local environmental variables explained 56.3% of the variance of the overall species richness, largely accounted for by regional factors (40.6%) and with bryophyte cover of branches (BCov; 13.3%) as the only important local factor (Figure 3). Epiphytic richness showed a very similar pattern with 55.4% of variance explained. Non-epiphytic richness had a similar total explained variance (55.2%), but with local factors (27.3%) equally important as the regional factors (27.8%). In particular, canopy height (HC 4.7%) and inclination (Inc 4.3%), which played essentially no role for the total and epiphytic richness, had important contributions for the non-epiphytes (Figure 3).

Regressing richness against Temp^2^ only had significant independent effects on total and epiphytic species richness (adjusted *R*^2^ = 0.15 and *R*^2^ = 0.24, respectively, *p* < 0.001 each). TempS accounted for 10% of variance explained (*p* < 0.01) in non-epiphytic richness, BCov only explained 16% and 34% of variance in total and non-epiphytic richness, respectively (*p* < 0.001; Supplementary Figure S1). The other variables had no noteworthy independent effects on species richness (*R*^2^ > 0.2 and *p* < 0.01).

Analyzing species richness based on the subplot records only resulted in higher explanatory power of the regional variables regarding the total species richness and the non-epiphytic species richness (56% and 33%), and almost similar explanatory power of the local variable set (Figure 3). Both variable sets performed better in explaining total epiphytic species diversity of the plots compared to the subplot records. The combined dataset performed roughly equally for the total species richness of the plots and the subplots, respectively. In contrast, the analyses of the subplot records decreased 7.9% of explained variance in epiphytic and non-epiphytic species richness with respect to the plot records. The relative importance of the local variables declined markedly in all three subplot-based species richness analyses compared to the plot-based diversity. Contrasting with this trend, the proportion of annual mean temperature (Temp + Temp^2^) increased.

## Discussion

The main result of our study is that both regional and local environmental factors influence liverwort richness in montane forests in Uganda, and that they interact in complex ways that affect epiphytic and non-epiphytic bryophytes differently. Similar interactions of regional and local factors have also been found for other plant groups such as ferns (Weigand et al., 2019).

Specifically, we found that total and epiphytic liverwort diversity was largely influenced by regional climatic factors, whereas non-epiphytic diversity was accounted for to similar degrees by regional and local factors.

The distribution of overall liverwort diversity was largely driven by regional climatic factors (temperature and precipitation, and their seasonality) and air humidity (as indicated by epiphytic bryophyte cover; Karger et al., 2012), so that ultimately temperature- and humidity-related variables had roughly equal contributions to total variance explained. The importance of these climatic factors in explaining geographical patterns of species richness in bryophytes is well known (Aranda et al., 2014; Medina et al., 2014; Chen et al., 2015), and can be linked to physiological limitations imposed by high temperatures and low humidity at low latitudes and elevations, and low temperatures at high latitudes and elevations (Rütten and Santarius, 1992; Leon-Vargas et al., 2006). The concordance of overall and epiphytic diversity patterns suggests that the overall pattern of species richness in liverworts in our study region was largely driven by the epiphytic species, which is unsurprising considering that they contributed 74.8% of the total species richness. Terrestrial bryophytes are scarce especially in tropical lowlands, where they are restricted to small patches of open soil (Richards, 1954; Pócs, 1982; Holz et al., 2002).

It is well known that especially epiphytic bryophyte communities are also influenced by other factors such as the characteristics of their host trees, for instance bark texture and pH or trunk diameter (Studlar, 1982; González-Mancebo et al., 2003; Gradstein and Culmsee, 2010). Our study did not assess these factors, but we do not consider that they strongly influence our results as our sampling strategy covered numerous trees at each site and elevation. As the forests studied by us are typically dominated by one or a few tree species (see the section “Materials and Methods”), our sampling covered these species. Since our study was focused on natural forests, the tree composition of the forests was determined by the combination of edaphic and climatic conditions of the study sites. Accordingly, even though tree-specific associations of the bryophytes were not included directly by us, their effect is to some degree indirectly covered by the environmental data included in the analyses.

In contrast to the overall pattern, non-epiphytic liverwort richness showed a quite distinct pattern, with a higher contribution of local environmental factors than epiphytes. This difference in epiphytic and non-epiphytic patterns likely reflects the different influences affecting their growth, in particular that epiphytic liverworts are more closely linked to climatic conditions (Medina et al., 2014), whereas non-epiphytic liverworts also depend on the local soil and microclimatic conditions. Both the impact of the amount of bare soil as well as site-specific climate conditions on non-epiphytic bryophyte species richness has been documented earlier (Pharo and Beattie, 2002; Mandl et al., 2009). Specifically, our analysis shows important effects of canopy height and slope inclination. The relationship to canopy height likely reflects the fact that tall forests at lower elevations provide highly unsuitable conditions for non-epiphytic bryophytes, with low light incidence at ground levels (Bazzaz and Pickett, 1980), and high accumulation of leaf litter (Richards, 1954). In the lowlands and lower montane forests, terrestrial species thus almost exclusively occur on disturbed soil surfaces like termite hills and earth walls (Richards, 1954; Pócs, 1982), which are formed by digging activities of animals or thrown up by uprooted trees (Jonsson and Esseen, 1990). Slope inclination is important for bryophytes because steeper slopes, depending on the soil properties, have more and larger canopy gaps created by uprooting events (Ohkubo et al., 2007; de Lima and de Moura, 2008), and hence, provide increased light exposure and patches of bare soil as well as dead wood.

Comparing the resulting RVI with the univariate regressions of the respective variables furthermore indicates that the balance of co-acting effects of all variables in the models are determinant for the richness patterns in diversity.

Focusing on the sampling strategy, we found that our standardized sampling strategy with small subplots included the majority of species present in each plot. Generally speaking, the results obtained by including only species recorded in the subplots versus including also the additionally recorded species in the entire plot were largely concordant. There was one minor, but potentially interesting difference, however: when we only included the species recorded in the subplots, the importance of local factors diminished in the GLM analyses. We interpret this as reflecting that the subplots predominantly covered ecologically generalist species, as 72.7% of the omitted species from the total plot collection were found in only one plot. Thus, the declining importance of local predictors for the subplot diversity probably reflects the good adaptability of the more widely distributed species and neglects the less widely distributed species with narrower environmental tolerance included in the plot-based analyses. It would appear that the latter species are more dependent on local microhabitat conditions.

Our study therefore confirms that the richness of epiphytic liverworts is closely linked to regional climatic conditions, whereas the occurrence of non-epiphytic liverworts depends much more on local microhabitat-specific factors. This implies, among other aspects, that species distribution models, which generally largely rely on macroclimatic factors (Lembrechts et al., 2019), would be expected to perform well for epiphytic bryophytes, but much more poorly for non-epiphytic and rare ones. For the latter, local-scale factors, which are generally not available in macroecological studies, would be needed to accurately predict their probabilities of occurrence. As already suggested by Zotz and Bader (2009) it may also be hypothesized that epiphytic bryophyte richness may react more directly to changes in regional climate in the course of global change than non-epiphytic richness, which may more strongly depend on the availability of small-scale microrefugia (Mandl et al., 2009; Raabe et al., 2010; Sun et al., 2013).

## Data Availability Statement

All datasets generated for this study are included in the article/Supplementary Material.

## Author Contributions

KM, MK, and DQ designed this study. DQ, B-CH, KM, and MK conducted the field work. DQ, KM, and B-CH designed the set-up of the collection. B-CH, DQ, and KM collected the bryophyte specimens. MK recorded the local parameters of the plots. Y-MW identified the leafy liverworts. MN identified the thallose liverworts. B-CH processed the specimens and associated the labels. KM performed the analyses and wrote the manuscript. FL contributed to and revised the statistical analyses. All co-authors provided critical comments on the manuscript and have read and approved the submitted manuscript.

## Conflict of Interest

The authors declare that the research was conducted in the absence of any commercial or financial relationships that could be construed as a potential conflict of interest.
