# Supplementary material for: Different Predictors Shape the Diversity Patterns of Epiphytic and Non-epiphytic Liverworts in Montane Forests of Uganda
Source: Front Plant Sci. 2020 Jun 24;11:765. doi: 10.3389/fpls.2020.00765 (PMC7327462; doi:10.3389/fpls.2020.00765)
Supplement: Supplementary file 1 [file Table_1.docx]

**Table S1:** **Species numbers, regional and local factors of the study plots.** Rad; solar radiation (Wilson and Gallant, 2000); Temp; annual mean temperature; Temp^2^, (annual mean temperature)^2^; TempS, temperature seasonality; Prec, annual precipitation; PrecS, precipitation seasonality (Karger et al., 2017); BCov, bryophyte cover of branches [%]; HC, canopy height [m]; Inc, inclination; GCov, ground covered by plants [%]; CCov, canopy cover [%]; DW, distance to open water [m].

| **Plot** | **All** | **Epiphytes** | **Non-Epiphytes** | **All** | **Epiphytes** | **Non-Epiphytes** | **Rad** | **Temp** | **Temp²** | **TempS** | **Prec** | **PrecS** | **BCov** | **HC** | **Inc** | **GCov** | **CCov** | **DW** |
| --- | --- | --- | --- | --- | --- | --- | --- | --- | --- | --- | --- | --- | --- | --- | --- | --- | --- | --- |
|  | Plot records | | | Subplot records only | | |  |  |  |  |  |  |  |  |  |  |  |  |
| 1 | 22 | 15 | 10 | 18 | 12 | 9 | 5917.268066 | 193.0166626 | 37255.43204 | 354.3712769 | 1467 | 41.66044617 | 50 | 22 | 5 | 90 | 75 | 2800 |
| 2 | 19 | 17 | 3 | 17 | 15 | 3 | 5906.259766 | 194.3119049 | 37757.11639 | 354.4651489 | 1421 | 41.27931595 | 20 | 18 | 0 | 30 | 70 | 2800 |
| 3 | 12 | 9 | 4 | 8 | 8 | 1 | 5918.291504 | 208.5976257 | 43512.96945 | 372.1170654 | 1192 | 42.45198441 | 10 | 30 | 0 | 30 | 80 | 500 |
| 4 | 10 | 9 | 2 | 8 | 7 | 2 | 5910.438477 | 207.8238068 | 43190.73467 | 370.2541504 | 1251 | 42.84117508 | 30 | 30 | 5 | 75 | 50 | 20 |
| 5 | 21 | 18 | 9 | 16 | 15 | 3 | 5932.129883 | 197.5904846 | 39041.9996 | 361.5861206 | 1369 | 43.53218079 | 40 | 25 | 8 | 50 | 80 | 50 |
| 6 | 8 | 6 | 2 | 7 | 6 | 1 | 5842.769043 | 237.6166687 | 56461.68124 | 412.3691101 | 1117 | 33.95968246 | 1 | 25 | 0 | 50 | 70 | 50 |
| 7 | 8 | 7 | 1 | 7 | 6 | 1 | 5840.477051 | 237.6166687 | 56461.68124 | 412.3691101 | 1117 | 33.95968246 | 1 | 30 | 0 | 70 | 75 | 200 |
| 8 | 5 | 5 | 0 | 3 | 3 | 0 | 5842.440918 | 237.8833313 | 56588.47931 | 418.0296326 | 1126 | 33.89097595 | 1 | 25 | 0 | 30 | 75 | 600 |
| 9 | 26 | 22 | 13 | 19 | 14 | 11 | 5838.300293 | 195.5785828 | 38250.98205 | 493.6977844 | 1008 | 31.17669106 | 100 | 25 | 5 | 50 | 90 | 0 |
| 10 | 27 | 17 | 15 | 22 | 15 | 12 | 5799.786133 | 195.5785828 | 38250.98205 | 493.6977844 | 1008 | 31.17669106 | 95 | 25 | 10 | 40 | 85 | 30 |
| 11 | 28 | 21 | 10 | 28 | 21 | 9 | 5836.180664 | 191.4214325 | 36642.16482 | 482.0240479 | 1174 | 31.23220253 | 90 | 25 | 15 | 40 | 85 | 20 |
| 12 | 16 | 14 | 3 | 11 | 9 | 2 | 5629.765137 | 195.9523773 | 38397.33417 | 493.1712341 | 1005 | 31.35370636 | 95 | 30 | 25 | 30 | 90 | 10 |
| 13 | 25 | 19 | 10 | 18 | 12 | 9 | 5885.678223 | 173.1238098 | 29971.85352 | 490.3063965 | 1070 | 34.11765289 | 100 | 30 | 45 | 30 | 90 | 30 |
| 14 | 23 | 20 | 8 | 22 | 19 | 8 | 5962.319824 | 173.1238098 | 29971.85352 | 490.3063965 | 1070 | 34.11765289 | 100 | 25 | 40 | 50 | 75 | 70 |
| 15 | 13 | 8 | 7 | 11 | 6 | 7 | 5807.15332 | 159.2809448 | 25370.41938 | 499.1064148 | 1370 | 39.47090149 | 100 | 15 | 15 | 80 | 95 | 450 |
| 16 | 3 | 3 | 2 | 3 | 3 | 0 | 5844.354492 | 156.0714264 | 24358.29014 | 493.5549927 | 1410 | 37.91534805 | 100 | 22 | 25 | 70 | 85 | 250 |
| 17 | 14 | 10 | 9 | 13 | 8 | 8 | 5921.117188 | 156.4523773 | 24477.34636 | 484.8354492 | 1283 | 36.63148117 | 100 | 15 | 20 | 60 | 95 | 200 |
| 18 | 16 | 13 | 9 | 11 | 9 | 4 | 5964.057129 | 163.2881012 | 26663.00399 | 494.6264038 | 1128 | 35.94264984 | 100 | 30 | 20 | 98 | 50 | 0 |
| 19 | 22 | 12 | 15 | 18 | 11 | 11 | 5828.844727 | 158.0833282 | 24990.33865 | 487.2125244 | 1275 | 35.34349442 | 100 | 30 | 15 | 85 | 60 | 100 |
| 20 | 12 | 8 | 4 | 7 | 6 | 2 | 5552.765137 | 129.430954 | 16752.37185 | 464.8538513 | 1629 | 36.58001328 | 95 | 10 | 15 | 95 | 80 | 2000 |
| 21 | 18 | 9 | 14 | 10 | 8 | 6 | 4705.350098 | 100.0976181 | 10019.53315 | 417.7221985 | 1744 | 37.25917435 | 100 | 8 | 45 | 100 | 80 | 2000 |
| 22 | 7 | 6 | 2 | 5 | 5 | 1 | 5675.18457 | 129.430954 | 16752.37185 | 464.8538513 | 1629 | 36.58001328 | 100 | 6 | 35 | 100 | 90 | 2000 |
| 23 | 22 | 19 | 6 | 17 | 17 | 3 | 5988.64502 | 129.430954 | 16752.37185 | 464.8538513 | 1629 | 36.58001328 | 100 | 12 | 5 | 100 | 95 | 2000 |
| 24 | 15 | 7 | 11 | 12 | 5 | 8 | 5945.657227 | 129.430954 | 16752.37185 | 464.8538513 | 1629 | 36.58001328 | 100 | 18 | 20 | 50 | 95 | 2000 |
