# Supplementary material for: Different Predictors Shape the Diversity Patterns of Epiphytic and Non-epiphytic Liverworts in Montane Forests of Uganda
Source: Front Plant Sci. 2020 Jun 24;11:765. doi: 10.3389/fpls.2020.00765 (PMC7327462; doi:10.3389/fpls.2020.00765)
Supplement: Supplementary file 2 [file Data_Sheet_1.docx]

**List S1: References used for species identification**

**Books**

**Arnell, S.W. 1963**. *Hepaticae of South Africa*. Stockholm.

**Fischer, E. 2013.** *Liverworts and Hornworts of Rwanda*. Abc Taxa **14**: 1-552. Brussels.

**Sim, T.R. 1926.** The Bryophyta of South Africa. Transactions of the Royal Society of South Africa 15: 1-475

**Wigginton, M.J. (ed.) 2004**. E.W. Jones’s Liverwort and Hornwort Flora of West Africa. National Botanic Garden of Belgium, 443pp.

**Articles**

**Arnell, S.W. 1952.** Hepaticae collected in South and West Africa, 1951. New and little-known species. *Botaniska Notiser* **105**: 307-329.

**Arnell, S.W. 1953a.** Notes on South African Hepaticae. *Revue Bryologique et Lichénologique* **22**: 3-5.

**Arnell, S.W. 1953b.** Hepaticae collected in South Africa 1951. New and little known species. III. *Botaniska Notiser* **106**: 271-289.

**Arnell, S.W. 1955.** Notes on South African Hepatics II. *Botaniska Notiser* **108**: 309-313.

**Arnell, S.W. 1956a**. Hepaticae collected by O. Hedberg *et al.* on the East African mountains. *Arkiv för Botanik, ser. 2*. **3**: 517-562.

**Arnell, S.W. 1956b.** Hepaticae collected by K. Bystrøm in Fernando Po and Annobón, West Africa. *Svensk Botanisk Tidskrift* **50**: 527-534.

**Arnell, S.W. 1957a.** Notes on some African Hepaticae IV. *Botaniska Notiser* **110**: 17-27.

**Arnell, S.W. 1957b.** Notes on some South African Hepatics V. *Botaniska Notiser* **110**: 399-405.

**Arnell, S.W. 1957c.** Hepaticae collected in South-West Africa by Prof. Dr. O.H. Volk. *Mitteilungen der Botanischen Staatssammlung München* **16**: 262-272.

**Arnell, S.W. 1958a.** New Hepaticae from Cameroon Mountain. *Svensk Botanisk Tidskrift* **52**: 63-67.

**Arnell, S.W. 1958b.** Genus *Jungermannia* in South Africa. *Botaniska Notiser* **111**: 619-622.

**Arnell. S.W. 1960.** Hepatics collected by Dr John Eriksson in Abyssinia in 1958. *Svensk Botanisk Tidskrift* **54**: 187-192.

**Arnell, S.W. 1961a.** Hepatics collected in the Cape Verde Islands by Mr Knut Bystrøm. *Botaniska Notiser* **114**: 176-180.

**Arnell, S.W. 1961b.** Notes on South African Hepaticae VI. *Botaniska Notiser* **114**: 400-402.

**Arnell, S.W. 1962a.** Notes on South African Hepaticae VII. *Botaniska Notiser* **115**: 203-207.

**Arnell, S.W. 1962b.** Hepatics collected by Dr G. Degelius in Angola and Congo in 1960. *Svensk Botanisk Tidskrift* **56**: 55-60.

**Arnell, S.W. 1965.** Hepaticae collected by Mr Gillis Een in Mauritius and Réunion in 1962. *Svensk Botanisk Tidskrift* **59**: 65-84.

**Fischer, E. 1993.** Taxonomic results of the BRYOTROP Expedition to Zaire and Rwanda. 10. Trichocoleaceae, Geocalycaceae, Acrobolbaceae, Balantiopsidaceae, Lepidoziaceae (*Telaranea, Arachniopsis*), Calypogeiaceae, Adelanthaceae, Porellaceae, Jubulaceae, Marchantiaceae (*Dumortiera*), Polytrichaceae. *Tropical Bryology* **8**: 83-98.

**Fischer, E. 1999.** A new species of *Colura* (Lejeuneaceae) from the Aberdare Mountains, Kenya. *Tropical Bryology* **16**: 205-208.

**Fischer, E. & Thiel, C. 2015.** New and noteworthy records of liverworts (Marchantiopsida) and hornworts (Anthocerotopsida) for Rwanda. *Nova Hedwigia* **100**: 525-534.

**Gradstein, S.R. & Vanden Berghen, C. 1985.** *Schiffneriolejeunea* sect. *Pappeanae* en Afrique. *Beihefte zur Nova Hedwigia* **80**: 173-193.

**Jones, E.W. 1953a.** African Hepatics II. *Leptocolea* with hyaline-margined leaves. *Transactions of the British Bryo-logical Society* **2**: 144-157.

**Jones, E.W. 1953b.** African Hepatics III. *Cololejeunea* and *Leptocolea* with dentate leaves. *Transactions of the British Bryological Society* **2**: 158-163.

**Jones, E.W. 1953c.** African Hepatics V. *Lophocolea*, with notes on *Chiloscyphus* and *Leptoscyphus*. *Transactions of the British Bryological Society* **2**: 172-202.

**Jones, E.W. 1954a.** African Hepatics VI. *Euosmolejeunea*. *Transactions of the British Bryological Society* **2**: 375-379.

**Jones, E.W. 1954b.** African Hepatics VII. The genus *Cheilolejeunea*. *Transactions of the British Bryological Society* **2**: 380-392.

**Jones, E.W. 1954d.** African Hepatics IX. Some species of *Ptychocoleus*. *Transactions of the British Bryological Soci-ety* **2**: 396-407.

**Jones, E.W. 1954e.** African Hepatics X. *Leptocolea* and *Cololejeunea*. *Transactions of the British Bryological Society* **2**: 408-438.

**Jones, E.W. 1957a.** African Hepatics XII. Some little-known Lejeuneaceae. *Transactions of the British Bryological Society* **3**: 191-207.

**Jones, E.W. 1958.** A provisional key to the genera of tropical African hepatics. *Journal of the West African Science Association* **4**: 50-73.

**Jones, E.W. 1962.** African Hepatics XV. *Plagiochila* in Tropical Africa. Transactions of the British Bryological Socie-ty 4: 254-325.

**Jones, E.W. 1963.** African Hepatics XVI. *Porella* in Tropical Africa. Transactions of the British Bryological Society 4: 446-461.

**Jones, E.W. 1967.** African Hepatics XVIII. *Taxilejeunea*, and *Lejeunea* with eplicate perianths. *Transactions of the British Bryological Society* **5**: 289-304.

**Jones, E.W. 1968a.** African Hepatics XIX. The *Lejeunea flava* complex. *Transactions of the British Bryological Soci-ety* **5**: 548-562.

**Jones, E.W. 1968b.** African Hepatics XX. Some little-known species and extensions of range. *Transactions of the British Bryological Society* **5**: 563-572.

**Jones, E.W. 1969.** African Hepatics XXI. *Microlejeunea, Chaetolejeunea* and *Pleurolejeunea*. *Transactions of the British Bryological Society* **5**: 775-789.

**Jones, E.W. 1972.** African Hepatics XXIII. Some species of *Lejeunea*. *Journal of Bryology* **7**: 23-45.

**Jones, E.W. 1974a.** African Hepatics XXIV. Lejeuneaceae: some new or little-known species, and extensions of range. *Journal of Bryology* **7**: 545-561 "1973".

**Jones, E.W. 1974b.** African Hepatics XXV. *Rectolejeunea*. *Journal of Bryology* **8**: 71-75.

**Jones, E.W. 1974c.** African Hepatics XXVI. The *Lejeunea eckloniana* complex. *Journal of Bryology* **8**: 77-91.

**Jones, E.W. 1975.** African Hepatics XXVII. *Bazzania*. *Journal of Bryology* **8**: 299-316.

**Jones, E.W. 1976a.** African Hepatics XXVIII. *Schistochila* Dumort. *Journal of Bryology* **9**: 33-41.

**Jones, E.W. 1976b.** African Hepatics XXIX. Some new or little-known species, and extensions of range. *Journal of Bryology* **9**: 43-54.

**Jones, E.W. 1977.** African Hepatics XXX. The genus *Radula* Dumortier. *Journal of Bryology* **9**: 461-504.

**Jones, E.W. 1979.** African Hepatics XXXI. Rare or little-known Lejeuneaceae and extensions of range. *Journal of Bryology 10: 387-400.*

**Jones, E.W. 1981.** *African Hepatics XXXII. Some little-known species and extensions of range. Journal of Bryology 11: 311-323. "1980*".

**Jones, E.W. 1985a.** African Hepatics XXXIV. Little-known or new Lejeuneaceae. *Journal of Bryology* **13**: 385-398.

**Jones, E.W. 1985b.** African Hepatics XXXV. Some new or little-known species and noteworthy extensions of range. *Journal of Bryology* **13**: 497-508.

**Jones, E.W. 1988.** African Hepatics XXXVIII. *Cheilolejeunea* subgen *Strepsilejeunea* (Spruce) Schuster with special reference to East Africa. *Journal of Bryology* **15**. 149-160.

**Jones, E.W. 1989.** African Hepatics XXXIX. Some dioecious species of *Lejeunea*. *Journal of Bryology* **15**: 665-673.

**Jones, E.W. & Harrington, A.J. 1983.** The Hepaticae of Sierra Leone and Ghana. *Bulletin of the British Museum* (*Natural History*)*, Botanical series* **11**: 215-289.

**Jovet-Ast, S. 1946.** Hépatiques des Iles du Cap Vert. *Mémoires de la Société de Biogéographie* **8**: 363-367.

**Jovet-Ast, S. 1954.** Le genre *Colura*, Hépatiques, Lejeuneacées, Diplasiae. *Revue Bryologique et Lichénologique* **22**: 206-312.

**Jovet-Ast, S. & Bischler, H. 1971.** Les Hépatiques d'Egypt et du Sinai. Enumération, notes écologiques et biogéographiques. *Revue Bryologique et Lichénologique* **37**: 265-290.

**Pócs, T. 1975.** New or little-known epiphyllous liverworts I. *Cololejeunea* from tropical Africa. *Acta Botanica Aca-demiae Scientiarum Hungaricae* **21**: 353-375.

**Pócs, T. 1993b.** Taxonomic results of the BRYOTROP Expedition to Zaire and Rwanda. 12. Metzgeriaceae, Plagi-ochilaceae, Lejeuneaceae (the non-epiphyllous collections). *Tropical Bryology* **8**: 105-126.

**Pócs, T. 1994b.** Taxonomic Results of the BRYOTROP Expedition to Zaire and Rwanda. 27. Lepidoziaceae, II. *Trop-ical Bryology* **9**: 123-130.

**Pócs, T. 1994c.** Taxonomic Results of the BRYOTROP Expedition to Zaire and Rwanda. 28. Lejeuneaceae, a ramicolous collection. *Tropical Bryology* **9**: 131-136.

**Pócs, T. 1994d.** East African Bryophytes, XIII. Bryophytes from the Bale Mountains, S.E. Ethiopia. 2. *Hepaticae. Fragmenta Floristica et Geobotanica 39: 221-233.*

**Pócs, T. 1995.** *East African Bryophytes, XIV. Hepaticae from the Indian Ocean Islands. Fragmenta Floristica et Geo-botanica 40: 251-277.*

**Pócs, T. 2001.** *East African Bryophytes*, XVI. New taxa of Lejeuneoideae (Lejeuneaceae) collected in Manongarivo Special Reserve, NW Madagascar. *Candollea* **56**: 69-78.

**Pócs, T. 2010a.** On some less known *Lejeunea* (Lejeuneaceae, Jungermanniopsida) species in tropical Africa. East African Bryophytes, XXVII. *Nova Hedwigia, Beihefte* **138**: 99–116.

**Pócs, T. 2011a.** Type studies of some African Lejeuneaceae. *Acta botanica hungarica* **53**: 181-192.

**Pócs, T. 2011b.** New or little known epiphyllous liverworts, XIV. The genus *Colura* (Lejeuneaceae) in SãoTomé Is-land, with the description of *Colura thomeensis* sp. nov. *The Bryologist* **114**: 362-366.

**Pócs, T. 2011c.** East African Bryophytes XXIX. The Ceratolejeunea (Lejeuneaceae) species of the Indian Ocean Is-lands. Polish Botanical Journal 56: 131-153.

**Pócs, T. & Luke, Q. 2007.** East African Bryophytes, XXV. Bryological records from the Chyulu Range, Kenya. *Jour-nal of East African Natural History* **96**: 27-46.

**Pócs, T. & Lye, K. 1999.** New records and additions to the hepatic flora of Uganda. 2. *Tropical Bryology* **17**: 23-33.

**Pócs, T, Ochyra, R. & Bednarek-Ochyra, H. 2016.** *Lepidozia cupressina* (Marchantiopsida, Lepidoziaceae) in sub-Saharan Africa, with a note on the taxonomic status of *L. chordulifera*. *Cryptogamie, Bryologie* **37**: 125-147.

**Pócs, T. & Sass-Gyarmati, A. 2006.** New or little known epiphyllous liverworts, XII. *Archilejeunea helenae* Pócs & Gyarmati, sp. nov. *Cryptogamie, Bryologie* **27**: 103-109.

**Pócs, T. & Váňa, J. 2015.** East African Bryophytes XXX. *Acta Biologica Plantarum Agriensis* **3:** 3-21.

So, M.L. (2004): *Metzgeria* (Metzgeriaceae, Marchantiophyta) in Africa. New Zealand Journal of Botany 42:271-292.

**Tixier, P. 1975.** Contribution à l'étude de l'hépaticologie africaine. I. Récoltes en bordure du Golfe de Guinée (Came-roun et Gabon). *Annales de la Faculté des Sciences de Yaoundé* **20**: 3-10.

**Tixier, P. 1976.** La notion d'èspece chez le genre *Cololejeunea*. Une epèce circumtropicale: *Cololejeunea filicaulis* St. *Revue bryologique et Lichénologique* **41**: 465-472.

**Tixier, P. 1977.** Espèces nouvelles malgaches du genre *Diplasiolejeunea* (Spruce) Schiffn. (Hepaticae). *Lindbergia* **4**: 117-125.

**Tixier, P. 1979a**. La famille des Cololejeuneoideae (Grolle) dans l'Ocean Indien Occidental - Essai monographique. *Bulletin de l'Academie Malgache* **55**(1-2): 173-247.

**Tixier, P. 1979b.** Contribution à l'étude du genre *Cololejeunea*. Les Cololejeunoidées de Nouvelle Calédonie. Essai monographique. *Nova Hedwigia* **31**: 721-787.

**Tixier, P. 1979c.** Nouvelles espèces Malgaches de *Diplasiolejeunea* (Diplasiae). *Revue bryologique et lichénologique* **45**: 209-226.

**Tixier, P. 1983.** Bryophyta Exotica. VII. Les Montagnes du Togo. Compléments bryologiques. *Annales de Université d'Abidjan, sér. C* (*Sciences*) **19**: 321-326.

**Tixier, P. 1984.** Contribution à l'étude du genre *Diplasiolejeunea* (Spruce) Schiffn. 4. La Section *Villaumeae* sur la côte est de Madagascar. *Acta Botanica Hungarica* **30**: 11-26.

**Tixier, P. 1985.** Contribution à la Connaissance des Cololejeuneoideae. *Bryophytorum Bibliotheca* **27**: 1-439.

**Tixier, P. 1989.** La notion de domaine pantropical. Le cas du genre *Cololejeunea*. *Compte Rendu des Séances de la Société Biogéographie* **65**: 175-179.

**Tixier, P. 1993.** Bryophytes nouveaux pour la science et l'Île Maurice. *Proceedings of the Royal Society of Arts and Sciences of Mauritius* **5**: 41-48.

**Tixier, P. 1995.** Résultats taxonomiques de l'éxpedition BRYOTROP au Zaire et Rwanda. 30. Bryophytes épiphylles (récoltes de E. Fischer). *Tropical Bryology* **11**: 11-76.

**Tixier, P. & Guého, J. 1997.** *Introduction to Mauritian Bryology. A check-list of mosses and liverworts*. Réduit: Mauritius Sugar Industry Research Institute.

**Vanden Berghen, C. 1946.** Note sur *Sprucella succida* (Mitt.) Steph. au Congo belge. *Bulletin du Jardin Botanique de l'État à Bruxelles* **18**: 89-95.

**Vanden Berghen, C. 1948a.** Note sur le genre *Ptychocoleus* Trevis. en Afrique tropicale. *Bulletin du Jardin Botanique de l'État à Bruxelles* **19**: 37-49.

**Vanden Berghen, C. 1948b.** Contribution á l'étude d'espèces africaine du genre *Metzgeria*. *Bulletin du Jardin Bota-nique de l'État à Bruxelles* **19**: 187-204.

**Vanden Berghen, C. 1948c.** Un nouveau genre d'Hépatiques *Evansiolejeunea* nov. gen. *Revue Bryologique et Li-chénologique* **17**: 86-90.

**Vanden Berghen, C. 1948d.** Notes sur quelques Lejeuneacées de l'Afrique centrale. *Revue Bryologique et Li-chénologique* **17**: 91-100.

**Vanden Berghen, C. 1949.** Contribution a l'étude des espèces africaines du genre *Mastigolejeunea* (Spruce) Schiffn. *Bulletin du Jardin Botanique de l'État à Bruxelles* **19**: 371-382.

**Vanden Berghen, C. 1950a.** Contribution à l'étude des espèces africaines du genre *Lopholejeunea* (Spruce) Schiffn. *Bulletin du Jardin Botanique de l'État à Bruxelles* **20**: 161-179.

**Vanden Berghen, C. 1950b.** Le genre *Thysananthus* en Afrique. *Revue Bryologique et Lichénologique* **19**: 35-37.

**Vanden Berghen, C. 1951a.** Contribution a l'étude des espèces africaines du genre *Ceratolejeunea* (Spruce) Schiffn. *Bulletin du Jardin Botanique de l'État à Bruxelles* **21**: 61-81.

**Vanden Berghen, C. 1951b.** Contribution a l'étude des espèces africaines du genre *Brachiolejeunea* (Spruce) Schiffn. *Bulletin du Jardin Botanique de l'État à Bruxelles* **21**: 87-94.

**Vanden Berghen, C. 1951c.** Contribution a l'étude des espèces africaines du genre *Archilejeunea* (Spruce) Schiffn. *Revue Bryologique et Lichénologique* **20**: 112-121.

**Vanden Berghen, C. 1952.** Notes sur quelques Lejeuneacées de l'Afrique Continentale. *Bulletin du Jardin Botanique de l'État à Bruxelles* **22**: 165-175.

**Vanden Berghen, C. 1953a.** Quelques hépatiques recoltées par O. Hedberg sur les montagnes de l'Afrique orientale. *Svensk Botanisk Tidskrift* **47**: 263-283.

**Vanden Berghen, C. 1953b.** Le genre *Leptolejeunea* (Spruce) Schiffn. en Afrique Continentale. *Bulletin du Jardin Botanique de l'État à Bruxelles* **23**: 65-72.

**Vanden Berghen, C. 1954.** *Le genre Marchantia L. au Congo Belge. Bulletin du Jardin Botanique de l'État à Bruxelles 24: 37-50.*

**Vanden Berghen, C. 1960a.** *Hépatiques récoltées par Dr J.-J. Symoens dans la région péri-tanganyikaise. Bulletin de la Société Royale de Botanique de Belgique* **92**: 111-138.

**Vanden Berghen, C. 1960b.** Hépatiques récoltées en Afrique par M. Monod. *Revue Bryologique et Lichénologique* **29**: 50-67.

**Vanden Berghen, C. 1961.** *Hépatiques récoltées par Dr J.-J. Symoens dans la région péri-tanganyikaise. Bulletin de la Société Royale de Botanique de Belgique 93: 55-74.*

**Vanden Berghen, C. 1963a***. Lejeunéacees* epiphylles d'Afrique. *Revue Bryologique et Lichénologique* **32**: 49-55.

**Vanden Berghen, C. 1963b.** Le genre *Nesolejeunea* Herz. *Revue Bryologique et Lichénologique* **32**: 292.

**Vanden Berghen, C. 1965.** Hépatiques récoltées par Dr J.-J. Symoens dans la région péri-tanganyikaise. *Bulletin de la Société Royale de Botanique de Belgique* **98**: 129-174.

**Vanden Berghen, C. 1972a.** Hépatiques épiphylles récoltées au Burundi par J. Lewalle. *Bulletin du Jardin Botanique National de Belgique* **42**: 431-494.

**Vanden Berghen, C. 1972b.** Hépatiques et Anthocérotées, in J.-J. Symoens, (ed.), *Résultats scientifiques. Exploration hydrobiologique du bassin du lac Bangweolo et du Luapula*. **8**(1): 1-220. Bruxelles: Cercle Hydrobiologique de Brux-elles.

**Vanden Berghen, C. 1973.** Quelques hépatiques récoltées au Gabon par G. le Testui. *Revue Bryologique et Li-chénologique* **39**: 365-385.

**Vanden Berghen, C. 1976.** Frullaniaceae (Hépaticae) Africanae. *Bulletin du Jardin Botanique National de Belgique* **46**: 1-220.

**Vanden Berghen, C. 1977.** Hépatiques épiphylles récoltées par J L De Sloover au Kivu (Zaïre), au Rwanda et au Bu-rundi. *Bulletin du Jardin Botanique National de Belgique* **47**: 199-246.

**Vanden Berghen, C. 1978a.** Notes sur quelques Lejeuneacees holostipées africaines. *Revue Bryologique et Li-chénologique* **44**: 123-132.

**Vanden Berghen, C. 1978b.** Hépatiques épiphylles récoltées au Rhodesie. *Revue Bryologique et Lichénologique* **44**: 443-452.

**Vanden Berghen, C. 1981.** Le genre *Plagiochila* à Madagascar et aux Mascarenes. *Bulletin du Jardin Botanique Na-tional de Belgique* **51**: 41-103.

**Vanden Berghen, C. 1983.** *Lepidozia* Dumort. emend. Joerg. subgen. *Sprucella* (Steph.) Vanden Berghen comb. et stat. nov. *Bulletin du Jardin Botanique National de Belgique* **53**: 321-330.

**Vanden Berghen, C. 1984a.** Le genre *Lopholejeunea* (Spruce) Schiffn. en Afrique. *Bulletin du Jardin Botanique Na-tional de Belgique* **54**: 393-464.

**Vanden Berghen, C. 1984b.** Le genre *Caudalejeunea* (Steph.) Schiffn. en Afrique. *Cryptogamie, Bryologie* **lichénol-ogie 5: 99-109.**

**Demaret, M. & Vanden Berghen, C. 1947.** Révision de quelques espèces africaines du genre *Frullania*. Bulletin du *Jardin Botanique de l'État à Bruxelles* 18: 231-240

**Wigginton, M.J., O'Shea, B.J., Porley, R.D. & Matcham, H.W. 2001.** Bryophytes of Uganda, 4. New and additional records, 2. *Tropical Bryology* **20**: 55-62.

**Yamada, K. 1993.** Taxonomic results of the BRYOTROP Expedition to Zaire and Rwanda. 13. Radulaceae. *Tropical Bryology* **8**: 127-130.

**Zwickel, W. 1933.** Zwei neue Gattungen, einige neue Arten und Umstellungen bei den Lejeuneaceen. *Annales Bryo-logici* **6**: 105-121.

**Taxonomic revisions and monographs**

**Gradstein, S.R. 1994.** *Flora Neotropica. Monograph no. 62. Lejeuneaceae: Ptychantheae, Brachiolejeuneae. New York: The New York Botanical Garden.*

**Gradstein, S.R. 2015.** *An overview of the genus Schiffneriolejeunea (Marchantiophyta: Lejeuneaceae). Nova Hed-wigia 100: 507-524.*

**Hodgetts, N.G. 2008.** *A morphological revision of the genus Herbertus S.Gray (Herbertaceae, Marchantiophyta) in Africa, including the East African Islands. Journal of Bryology 30: 239-263.*

**Malombe, I. 2007.** *Systematics of Cheilolejeunea (Spruce) Schiffn. (Lejeuneaceae) in continental Africa and its eco-logical signifiance* in Conservation of Kakamega and Budongo rainforests. Dissertation zur Erlangung des akad-emischen Grades eines Doktors der Naturwissenschaften Fachbereich 3: Mathematik/ Naturwissenschaften Universität Koblenz-Landau, 177 pp.

**Sukkharak, P. & Gradstein, S.R. 2014.** A taxonomic revision of the genus *Mastigolejeunea* (Marchantiophyta: Lejeuneaceae). *Nova Hedwigia* **99**: 279–345.
