# Supplementary material for: Different Predictors Shape the Diversity Patterns of Epiphytic and Non-epiphytic Liverworts in Montane Forests of Uganda
Source: Front Plant Sci. 2020 Jun 24;11:765. doi: 10.3389/fpls.2020.00765 (PMC7327462; doi:10.3389/fpls.2020.00765)

Figure S1: Graphs illustrate relationships between species richness and variables with highest relative importance. adj. R^2 = adjusted R^2, solid lines indicate Pr(>|z|) < 0.001, dotted lines = Pr(>|z|) < 0.05, dashed lines = Pr(>|z|) > 0.05. Not shown: Non-epiphytic richness ~ Height of canopy ($R^2 = 0$).

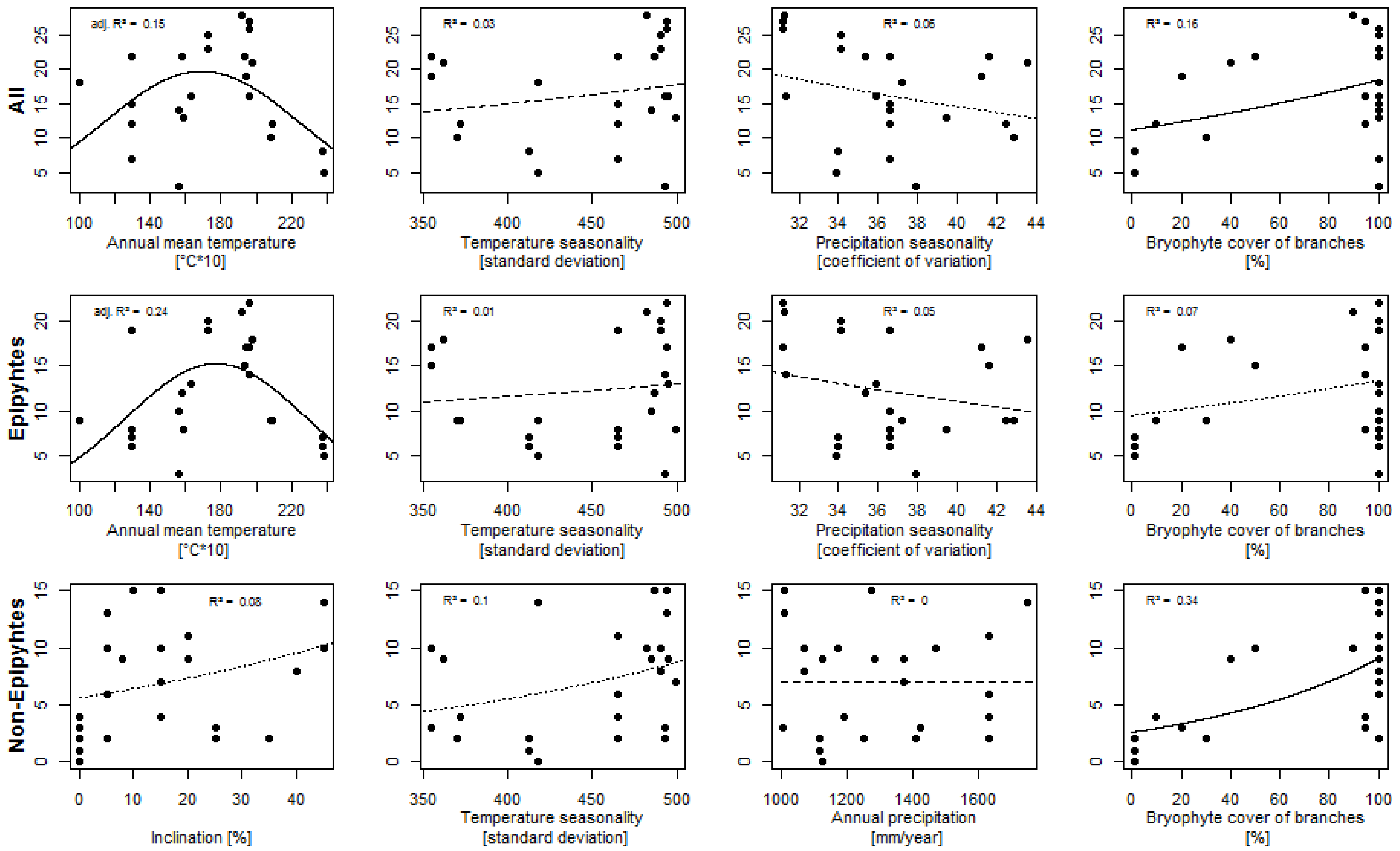

Supplement: Supplementary file 3 [file Image_1.pdf]
